# The Prognostic Implications of Tumor Infiltrating Lymphocytes in Colorectal Cancer: A Systematic Review and Meta-Analysis

**DOI:** 10.1038/s41598-020-60255-4

**Published:** 2020-02-25

**Authors:** Gregory E. Idos, Janet Kwok, Nirupama Bonthala, Lynn Kysh, Stephen B. Gruber, Chenxu Qu

**Affiliations:** 1 City of Hope National Medical Center, Duarte, CA USA; 2 University of Southern California Norris Comprehensive Cancer Center, Los Angeles, CA USA; 3 Cedars Sinai Medical Center, Los Angeles, California USA; 4 Institute of Nursing and Interprofessional Research (INIR), Children’s Hospital, Los Angeles, CA USA

**Keywords:** Cancer microenvironment, Colon cancer

## Abstract

Tumor-infiltrating lymphocytes (TILs) are an important histopathologic feature of colorectal cancer that confer prognostic information. Previous clinical and epidemiologic studies have found that the presence and quantification of tumor-infiltrating lymphocytes are significantly associated with disease-specific and overall survival in colorectal cancer. We performed a systematic review and meta-analysis, establishing pooled estimates for survival outcomes based on the presence of TILs in colon cancer. PubMed (Medline), Embase, Cochrane Library, Web of Science, and ClinicalTrials.gov were searched from inception to April 2017. Studies were included, in which the prognostic significance of intratumoral tumor infiltrating lymphocytes, as well as subsets of CD3, CD8, FOXP3, CD45R0 lymphocytes, were determined within the solid tumor center, the invasive margin, and tumor stroma. Random-effects models were calculated to estimated summary effects using hazard ratios. Forty-three relevant studies describing 21,015 patients were included in our meta-analysis. The results demonstrate that high levels of generalized TILS as compared to low levels had an improved overall survival (OS) with a HR of 0.65 (p = <0.01). In addition, histologically localized CD3+ T-cells at the tumor center were significantly associated with better disease-free survival (HR = 0.46, 95% CI 0.36–0.61, p = 0.05), and CD3 + cells at the invasive margin were associated with improved disease-free survival (HR = 0.57, 95% CI 0.38–0.86, p = 0.05). CD8+ T-cells at the tumor center had statistically significant prognostic value on cancer-specific survival and overall survival with HRs of 0.65 (p = 0.02) and 0.71 (p < 0.01), respectively. Lastly, FOXP3+ T-cells at the tumor center were associated with improved prognosis for cancer-specific survival (HR = 0.65, p < 0.01) and overall survival (HR = 0.70, p < 0.01). These findings suggest that TILs and specific TIL subsets serve as prognostic biomarkers for colorectal cancer.

## Introduction

Although advances in screening and treatment have substantially improved survival from colorectal cancer (CRC), clinical outcomes vary widely among patients with tumors diagnosed at the same TNM stage, and disease relapse occurs in 20–30% of patients with localized cancer^1^. The presence of microsatellite instability (MSI-H) in colorectal cancers have a better prognosis as compared to microsatellite stable (MSS) colorectal cancer^2–5^. The mechanisms that confer this benefit are not fully understood, but an association has been linked to the prominent infiltration of immune cells within the tumor^6^. Increased focus on the tumor microenvironment has identified inflammatory activity as a critical predictor of disease activity impacting patient prognosis.

The host immune response has been implicated in tumor behavior as it influences all phases of tumor development and growth^7–9^. Tumor-infiltrating lymphocytes (TILs) in histopathological analysis of CRC is often interpreted as the host protecting against tumor development^10,11^. TILs mediate recruitment, maturation, and activation of immune cells that suppress tumor growth. Tumor infiltration by T lymphocytes is a highly informative prognostic factor for CRC outcome, independent of traditional prognostic indicators^12–14^. Numerous studies have demonstrated that the type, density and site of tumor-infiltrating lymphocytes in primary tumors are prognostic for disease-free survival (DFS) and overall survival (OS) from CRC and hints at a fundamental function of the immune system in the tumor microenvironment^15–18^.

However, variability in study design, outcomes, sample size, and methods of measuring the host immune response reflecting the heterogeneity of studies in the literature inspired this systematic analysis. Recently, large retrospective studies have reported their data on the prognostic performance of TIL in CRC survival. To obtain a more precise estimate of the effect in populations with CRC, we performed an updated systematic review and meta-analysis to measure the impact of TILs on CRC survival.

## Methods

### Protocol and registration

We developed a protocol based on standard guidelines for the systematic review of prognostic studies and followed suggestions on updating systematic reviews as outlined by Moher *et al*.^19^. We followed the Preferred Reporting Items for Systematic Reviews and Meta-Analyses (PRISMA) statements for reporting our systematic review^20^. Methods of the analysis and inclusion criteria were specified *a priori* in a protocol.

### Data sources and search strategy

A librarian (LK) developed searches using a combination of keywords and controlled vocabulary (when available) in the following databases: PubMed (Appendix 1), Embase (Appendix 2 & 3), initially through OvidSP and later via Elsevier, Cochrane Library (Appendix 4), Web of Science (Appendix 5), and ClinicalTrials.gov (1997 to April 2017). In addition, we search grey literature sources (https//www.usa.gov, https://scholar.google.com) to identify relevant publications. The English language filter was used when available. We also examined bibliographies of related papers and reviews, while also consulting with experts in the field. In addition, we evaluated reference lists of previously published systematic reviews and meta-analysis.

### Eligibility criteria

All studies were reviewed initially based on title and abstract. If the data was insufficient based on title and abstract, the full text article would be reviewed. Two independent reviewers (GEI and NB) reviewed the first 100 results of the Ovid Medline search to assess for agreement of article selection with a kappa of 0.82. Then further search results were divided equally amongst GI and NB. Disagreement was resolved either by discussion, consensus or by a third party (SBG).

For study inclusion, the keywords included focused on generalized tumor inflammatory infiltrate and associated T lymphocytes’ subsets (CD4, CD8) in colorectal cancer patients identified with hematoxylin and eosin stain (HE) or immunohistochemical staining (IHC) and reported prognostic information. IHC staining was evaluated in subgroup analysis for tumor center (CC) and tumor stroma (TS) and at the invasive tumor margin (IM). Prognostic information included overall survival (OS) and disease-free survival (DFS).

Exclusion criteria included those publications for which there was insufficient data to estimate a hazard ratio (HR) with a 95% confidence interval (CI). However, references from review articles, case reports, commentaries and letter were reviewed to identify any additional studies that met the inclusion criteria. An effort was made to contact the authors for any clarifications.

### Data extraction and quality assessment

Two reviewers (GEI, JK) independently evaluated and extracted relevant information from each included study. We utilized a form originally developed from the work of McShane *et al*.^21^ and Hayes *et al*.^22^ adapted by Mei *et al*.^18^ for quality assessment in their systematic review and meta-analysis as this adaptation was comprehensive (See Supplement 1). It resulted in a quality rating of 0–9 based on reporting of inclusion and exclusion criteria, study design (prospective or retrospective), patient and tumor characteristics, description of the method or assay, study endpoints, follow-up time with patients and number of patients that dropped out during the follow-up period^18^.

### Data collection process

A standardized data abstraction form was adapted from Mei *et al*. to include key elements pertaining to the study design, sample size, patient age, stage of disease, assay method, follow-up duration, and HR estimates (with the corresponding 95% CIs) for TILs at certain locations within tumors (CT, TS or IM) and the HR cutoff point, method of quantifying (immunohistochemistry, PCR, sequencing). For time-to-event outcomes, we retrieved and curated the HR estimates with 95% CI from the original articles^18^. Discrepancies in interpretation between reviewers (GEI, NB) were resolved by discussion with a third reviewer (SBG) to reach consensus.

### Subgroup analyses

We performed analyses to estimate the association between prognostic outcomes (OS, CS, DFS) and both T-lymphocyte subsets (CD3 + , CD8 + , FOXP3 + , CD45R0 + ) and T lymphocyte infiltrate location (CC, TS, or IM). Survival time was recorded from either the date of diagnosis or the initiation of treatment, as available from the published reports. Random effects models were used consistent with prior published meta-analyses that showed evidence of heterogeneity for similar subgroup analyses.

### Summary measures

Meta-analyses were performed using the R package ‘meta’ version 4.9-0, using statistical software R (version 3.4.3). Random effects models were calculated based on HR estimates and their standard errors; inverse weighting was used for pooled variance. We then plotted forest and funnel charts by T-cell type, T-cell source and outcome to evaluate for publication bias. Interstudy heterogeneity was quantified using the *I*^2^ statistic, with an *I*^2^ value>50% as our *a priori* threshold for substantial heterogeneity^23^.

## Results

### Literature search

**Figure 1:**
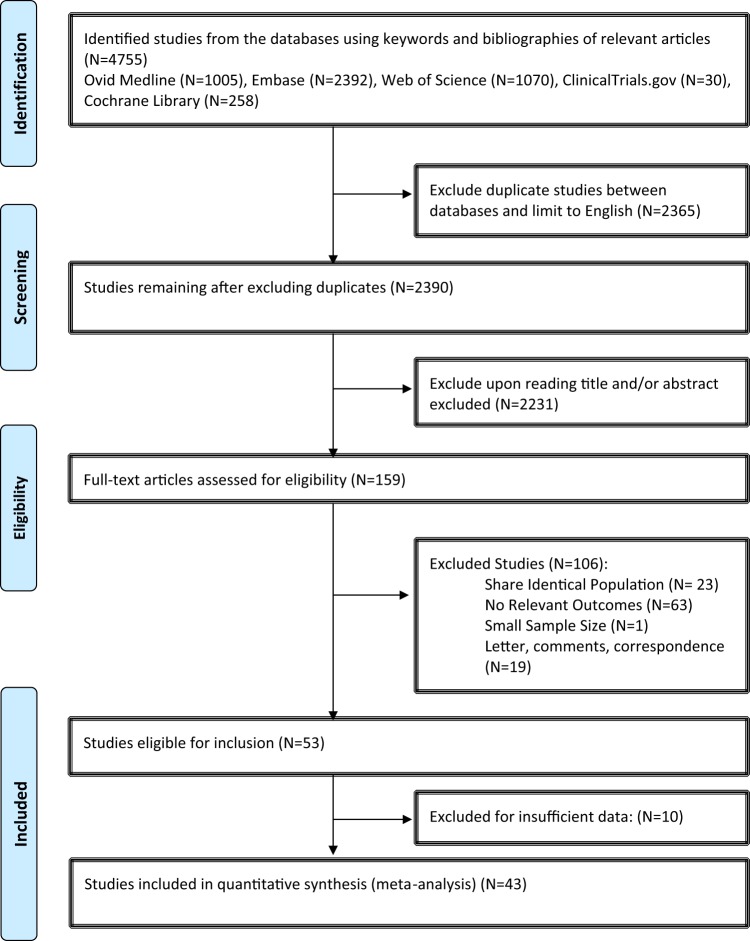
TIL Meta-Analysis Flow Diagram.

Eligible studies were identified and selected as shown in Fig. 1. Among the 3,789 studies identified for initial evaluation, 1,963 studies were eligible for further assessment based on pre-specified criteria. Abstracts of these studies were reviewed and 1,804 studies were excluded for the reasons delineated in Fig. 1. After abstract review, we identified 159 articles for full manuscript review and 106 of these studies were excluded. The most common reasons for exclusion were studies were the following: No relevant outcome (N = 63); Shared identical population (N = 23); and Editorial, letter, or commentary (N = 19). There were 53 studies eligible for inclusion, but 10 studies were found to have insufficient data. Therefore, 43 studies were included in the final meta-analysis (Table 1)^24–65^.

**Table 1:** Summary of study subsets and variables included in analysis.

Year	First Author	Counting Site	T-cell Subset	Outcomes	Assay	Variables
1997	Ropponen	General	TIL	OS	HE	Age, sex, site, surgical treatment, post-op complications, histology
1998	Naito	IM	CD8	OS	IHC	Pattern on invasion, histological type
1999	Nielsen	General	TIL	OS	IHC	Grade, site
2001	Guidoboni	CC	CD3, CD8, GRB	DFS, OS	IHC	Age, sex, pathologic stage, grade, histology, ploidy, adjuvant chemo, life status, recurrence
2001	Nagtegaal	General	CD3, CD4, CD8	OS	IHC/HE	N/A
2001	Paraf	CC	CD3	OS	IHC	Age, tumor size, expanding margin, CLR, tumor site, differentiation, pTNM stage, vascular and perineural invasion, peripheral adenomatous residue
2002	Cianchi	General	TIL	OS	Histopath	Age, sex, histotype, tumor differentiation, depth of invasion, venous invasion, character of invasive margin, conspicuous lymphocytic infiltration, tumor relapse
2004	Chiba	CC	CD8	CSS	IHC	Ki67, site, invasion pattern, differentiation
2004	Menon	IM	CD45	DFS	IHC/HE	Age, sex, location, stage, differentiation, mucinous, BM-like, recurrence
2004	Prall	CC	CD8	CSS, OS	IHC/HE	Location, substage, adjuvant therapy, MSI
2005	Buckowitz	General	TIL	OS	HE	Age, clinical criteria, treatment, localization, stage, T, N, M, Crohn’s like reaction, survival
2005	Gao	General	TIL	OS	HE	Gender, age, tumor location, Duke’s stage, growth pattern, differentiation, DNA ploidy, S-phase fraction, p53 expression
2005	Klintrup	General	TIL	OS	HE	Duke’s stage, WHO grade, tumor location
2006	Galon	CC	CD3 CT/IM pattern	OS	IHC	TNM, differentiation
2009	Ogino	General	TIL	CSS, OS	IHC	BMI, lymph node count, KRAS, BRAF, p53, PTGS2, MSI, CIMP, LINE-1 methylation
2009	Roxburgh	General	CD3	OS	IHC/HE	ki67, pi16, tumor differentiation, serosal involvement, margin involvement, tumor perforation, venous invasion, mGPS
2009	Salama	CC	FOXP3	CSS	IHC	Stage, tumor site, histologic grade, vascular invasion, lymphatic invasion, perineural invasion, lymphocytic response, MSI
2009	Sinicrope	TS	FoxP3	OS, DFS	IHC	Histologic grade, tumor site, chemo
2010	Correale	TS	CD8 CCR7+	OS	IHC/HE	Performance status, sex, age, tumor grade, liver mets
2010	Deschoolmeester	CC	CD3, CD8, GRB	OS, DFS	IHC	Location, grade, neo-adjuvant, adjuvant, MSI
2010	Frey	CC	CD3, CD8, FOXP3	CSS	IHC/HE	Age, tumor diameter, tumor location, grade, histology, vascular invasion, tumor border configuration
2010	Lee	TS	CD3, CD45RO, FOXP3	OS	IHC	CEA, size, lymphatic invasion, vascular invasion, perineural invasion
2010	Nosho	CC	CD3, CD8, CD45RO, FOXP3	OS, CSS	IHC	BMI, family history, tumor location, tumor grade, KRAS, BRAF, PIK3CA, MSI, CIMP, LINE-1 hypomethylation
2010	Peng	General	CD3, CD45RO	OS	IHC	Tumor site, pathologic grade
2010	Simpson	CC	CD3	CSS	IHC	Sex, grade, vascular invasion, site, MHC class I, MSI
2011	Dahlin	General	CD3 (MLH1, MSH2, MSH6, PMS2)	CSS	IHC	MSI
2012	Huh	General	TIL	OS, DFS	HE?	Age, tumor size, differentiation, lymphovascular invasion, perineural invasion, preoperative CEA, macroscopic ulceration, tumor border configuration
2012	Richards	General	CD3, CD8, CD45RO, FOXP3	CSS	HE stain	Age, sex, elective/emergency, tumor site, anemia, WBC, SIR(S), K-M, T, N, TNM, Peterson Index
2012	Yoon	CC	CD8	OS	IHC	Grade, site
2013	Kim	CC	FoxP3	OS	IHC	Age, gender, level of wall infiltration, lymph node metastasis
2013	Lewis	General	CLR (Crohns like reaction)	OS, PFS	HE	Lack of dirty necrosis, mucin differentiation, signet ring cell feature, medullary feature, histological heterogeneity, background dysplasia, 5-FU based chemo
2014	Di Caro	IM	CD3	DFS	IHC	N/A
2014	Ling	TS	CD8, FOXP3	CSS	IHC	MSI, CIMP
2014	Reimers	CC	Foxp3	OS, DFS	TMA, IHC	Age, gender, tumor grade, adjuvant therapy, circumferential margin
2014	Richards	IM	CD3, CD8, CD45RO, FOXP3	CSS	IHC	Preoperative systemic inflammatory response, Carstairs Deprivation Index, ASA grade, smoking status, POSSUM physiology scores, tumor differentiation, venous invasion, tumor necrosis, adjuvant chemo
2015	Kim	IM	CD8, CD45RO, FOXP3	OS	IHC, TMA	Age, gender, pTNM, lymphatic invasion, distant metastasis, MSI, CIMP, KRAS, BRAF, tumor location, adjuvant chemotherapy
2015	Mori	CC	CD8	DFS	IHC	NLR, PLR, CRP, MSI
2015	Reissfelder	CC	CD4, CD8, FOXP3	OS	IHC	Gender, UICC, TNM, operative procedure
2015	Vlad	IM	CD3	OS	IHC	Age, tumor location, TNM stage, histological grade, vascular, lymphatic and perineural invasion
2015	Wang	CC	FOXP3	OS	IHC	Age, gender, tumor size, differentiation, mucinous type, LN mets, T4, post-op chemo, tumor location, albumin
2016	Rozek	General	TIL, CLR	OS, CSS	HE	CLR, grade, MSI
2016	Sinicrope	General	FoxP3	DFS, OS	IHC	Histologic grade, tumor site, chemo
2016	Chen	CC	CD3, CD4, CD8, CD45R0,	DFS, OS	IHC	Age, gender, tumor site, TNM stage, LNR, VELIPI, tumor diameter, resection margin, differentiation, histopathology

### Study characteristics

Characteristics for each study are summarized in Table 1. Forty-three studies had a median quality score of 6 out of 9 (range: 3–8) and consisted of a median of 243 patients (range: 42–2,369), with a median follow-up of 64 months (range: 18–240). All studies were published from 1997–2017. There was one study included from an abstract due to the large number of patients (N = 2,293) included in the retrospective study (Sinicrope *et al*.^64^). Study sample sizes range from 42 to 2,396 patients representing an overall total of 21,015 patients. HRs and 95% CI for overall survival (OS), cancer-specific survival (CSS), or disease-free survival (DFS) were derived directly when available. A synopsis of study variables and results are summarized in Table 1 and Table 2, respectively.

**Table 2:** Summary of study outcome measures by subset.

	Location	Overall Survival	Cancer-Specific Survival	Disease-Free Survival
TIL	General	12 studies	4 studies	3 studies
		HR: 0.65 95% CI: 0.54–0.77	HR: 0.58 95% CI:0.46–0.73	HR: 0.72 95% CI:0.60–0.88
CD3	Total Studies	11 studies	4 studies	7 studies
	Tumor Center	HR: 0.67 95% CI:0.45–1.00	HR: 0.79 95% CI:0.57–1.11	HR: 0.46 95% CI:0.36–0.61
	Invasive Margin	HR: 0.69 95% CI:048–1.00	HR: 0.49 95% CI:0.38–0.63	HR: 0.57 95% CI:0.38–0.86
	Stroma	HR: 0.89 95% CI:0.49–1.61	HR: 0.58 95% CI:0.45–0.75	HR: 0.70 95% CI:0.27–1.81
CD4	Total Studies	2 studies		1 study
	Tumor Center	HR: 0.83 95% CI:0.53–1.30		HR: 0.55 95% CI:0.31–0.97
CD8	Total Studies	9 studies	5 studies	5 studies
	Tumor Center	HR: 0.71 95% CI: 0.53–0.94	HR: 0.65 95% CI:0.52–0.80	HR: 0.71 95% CI:0.53–0.94
	Invasive Margin	HR: 0.87 95% CI:0.71–1.07		HR: 0.61 95% CI:0.37–1.01
	Stroma	HR: 0.73 95% CI:0.56–0.97	HR: 0.71 95% CI:0.55–0.92	HR: 1.95 95% CI:0.66–5.76
CD45R0	Total Studies	5 studies	1 studies	2 studies
	Tumor Center	HR: 0.59 95% CI:0.45–0.78	HR: 0.13 95% CI:0.02–1.18	HR: 0.51 95% CI:0.33–0.80
	Invasive Margin	HR: 0.47 95% CI:0.33–0.66		
	Stroma	HR: 0.13 95% CI:0.02–1.16		HR: 0.20 95% CI:0.06–0.71
FoxP3	Total Studies	11 studies	4 studies	4 studies
	Tumor Center	HR: 0.70 95% CI:0.57–0.87	HR: 0.66 95% CI:0.55-0.79	HR: 0.75 95% CI:0.39–1.46
	Invasive Margin	HR: 0.65 95% CI:0.49–0.88	HR: 0.73 95% CI:0.56–0.96	
	Stroma	HR: 0.52 95% CI:0.27–0.99		HR: 0.48 95% CI:0.21–1.06
	General	HR:0.53 95% CI:0.24–1.18	HR: 0.65 95% CI:0.54–0.78	

### Subgroup analysis

Prognostic effect estimates were pooled for generalized tumor inflammatory infiltrate counts and T-lymphocyte subsets stratified by tumor location (IM, TS, CC) in CRC. Due to limited numbers and low sample sizes of studies within each subset, estimates of between-study heterogeneity were imprecise. Therefore, we performed funnel plot analyses for both generalized tumor inflammatory infiltrates and T-cell subsets.

### Generalized tumor infiltrating lymphocytes

**Figure 2:**
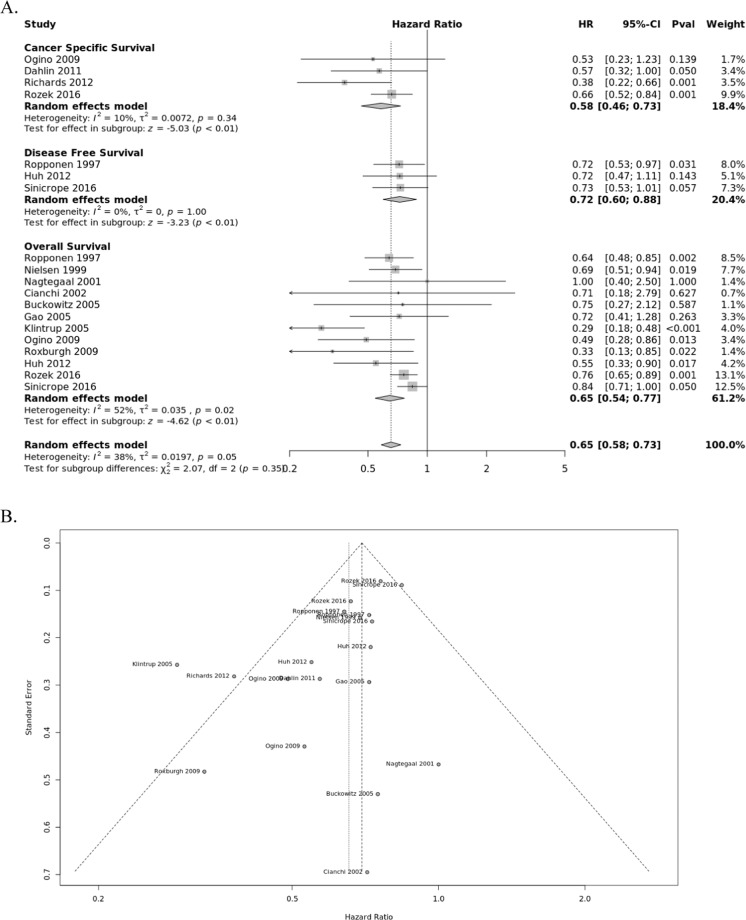
Forest plots of random effects between levels of generalized inflammatory infiltrate and survival. (**A**) The effect of generalized tumor infiltrate on cancer-specific survival (CSS), disease-free survival (DFS), and overall survival (OS). (**B**) Funnel plots of meta-analyses to assess the association between TILs and survival.

Density of generalized tumor infiltrates within CRC were pooled from fourteen studies for analysis (Fig. 2). All studies indicate improved prognosis for the presence of TILs for OS (HR = 0.65; 95% CI, 0.58–0.77), CSS (HR = 0.58; 95% CI, 0.46–0.73), and DFS (HR = 0.72; 95% CI, 0.60–0.88). There was no indication of publication bias for OS based on funnel plot analysis. However, moderate heterogeneity was noted in the OS subgroup (*I*^2^ = 54%, *P* = 0.02).

### CD3+ T lymphocyte subset

**Figure 3:**
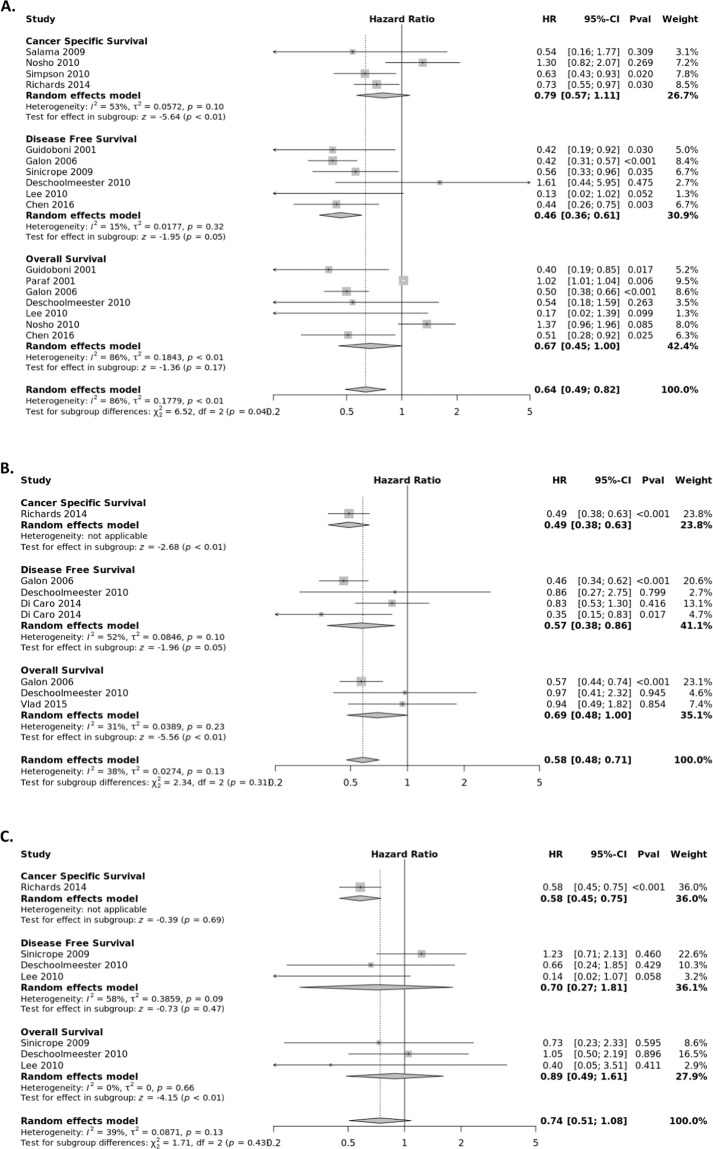
Forest plots of random effects between levels of CD3+ T-cell infiltrate and Survival. The effect of CD3+ T-cells in the (**A**) tumor center (**B**) invasive margin (**C**) stroma on cancer-specific survival (CSS), disease-free survival (DFS), and overall survival (OS).

The CD3 antigen is a T-cell co-receptor glycoprotein that plays an essential role in adaptive immune response. The association between the presence of CD3+ T lymphocytes and survival of CRC patients was extracted from fourteen studies (Fig. 3) stratified by tumor location, with eleven evaluating the tumor center, four the tumor stroma, and five the IM. The pooled HRs from the tumor center were calculated for OS (HR = 0.67; 95% CI, 0.45–1.00), CSS (HR = 0.79; 95% CI, 0.57–1.11), and DFS (HR = 0.46; 95% CI, 0.36–0.61). Statistically significant heterogeneity was observed between studies in the OS group (*I*^2^ = 86%, P < 0.01). The pooled HRs from the tumor margin (IM) were calculated for OS (HR = 0.69; 95% CI, 0.48–1.00), CSS (HR = 0.49; 95% CI, 0.38–0.63), and DFS (HR = 0.57; 95% CI, 0.38–0.86). The pooled HRs from the tumor stroma (TS) were calculated for OS (HR = 0.89; 95% CI, 0.49–1.61), CSS (HR = 0.58; 95% CI, 0.45–0.75), and DFS (HR = 0.70; 95% CI, 0.27–1.81).

### CD8+ T lymphocyte subset

**Figure 4:**
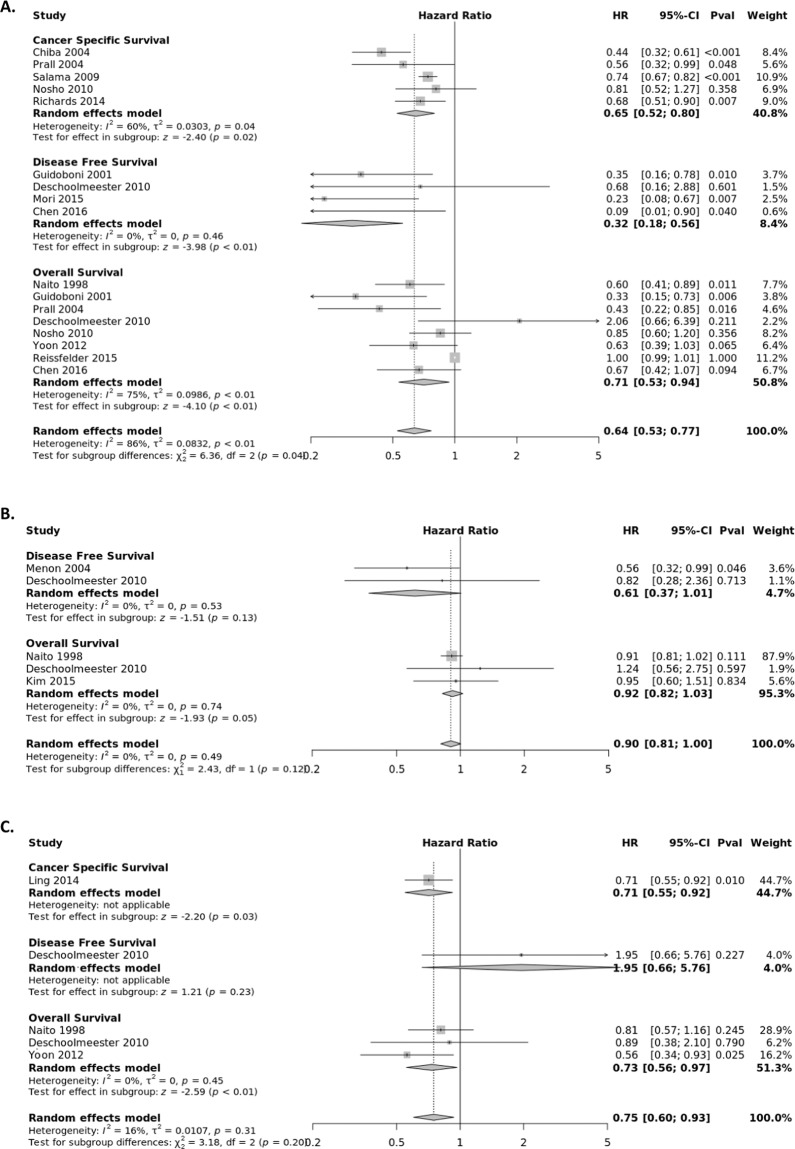
Forest plots of random effects between levels of CD8+ T-cell infiltrate and Survival. The effect of CD8+ T-cells in the (**A**) tumor center (**B**) invasive margin (**C**) stroma on cancer-specific survival (CSS), disease-free survival (DFS), and overall survival (OS).

CD8+ T cells are cytotoxic T cells that promote apoptosis of cancer cells^66^. The association between the presence of CD8+ T lymphocytes and survival of CRC patients was extracted from thirteen studies (Fig. 4) stratified by tumor location, with twelve evaluating the tumor center, five the stroma, and five the invasive margin. The pooled HRs from the tumor center were calculated for OS (HR = 0.71; 95% CI, 0.53–0.94), CSS (HR = 0.65; 95% CI, 0.52–0.80), and DFS (HR = 0.32; 95% CI, 0.18–0.56). Statistically significant heterogeneity was observed between studies for OS (*I*^2^ = 86%, P < 0.01). The pooled HRs from the IM were calculated for OS (HR = 0.92; 95% CI, 0.82–1.03) and DFS (HR = 0.61; 95% CI, 0.37–1.01). The pooled HRs from the TS were calculated for OS (HR = 0.73; 95% CI, 0.56–0.97) CSS (HR = 0.71; 95% CI, 0.55–0.92) and DFS (HR = 1.95; 95% CI, 0.66–5.76). Estimated HRs for CSS and DFS for CD8 + lymphocyte infiltrates from the tumor stroma were provided from a single study.

### FOXP3+ Treg subset

**Figure 5:**
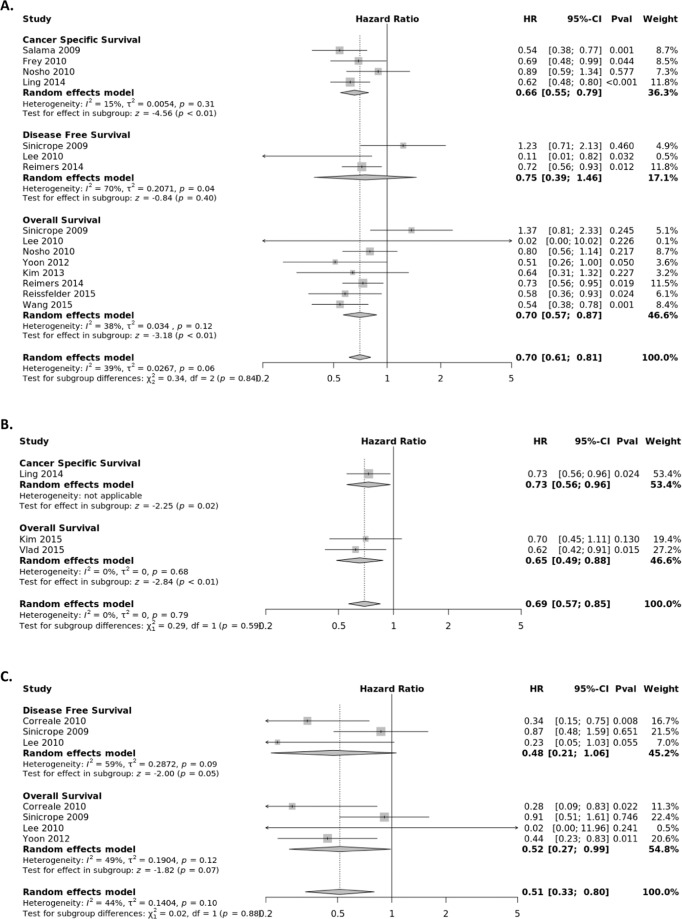
Forest plots of random effects between levels of FOXP3+ T-cell infiltrate and Survival. The effect of FOXP3+ T-cells in the (**A**) tumor center (**B**) invasive margin (**C**) stroma on cancer-specific survival (CSS), disease-free survival (DFS), and overall survival (OS).

FOXP3+ Tregs suppress aberrant immune response against self-antigens and maintain homeostasis of the immune system^67^. The association between the presence of FOXP3+ T lymphocytes and survival of CRC patients was extracted from fourteen studies (Fig. 5) stratified by tumor location, with eleven evaluating the CC, four the TS, and three the IM. The pooled HRs from the tumor center were calculated for OS (HR = 0.70; 95% CI, 0.57–0.87), CSS (HR = 0.66; 95% CI, 0.55–0.79) and DFS (HR = 0.75; 95% CI, 0.39–1.46). The pooled HRs from the IM were calculated for OS (HR = 0.65; 95% CI, 0.49–0.88) and CSS (HR = 0.73; 95% CI, 0.56–0.96). The pooled HRs from the TS were calculated for OS (HR = 0.52; 95% CI, 0.27–0.99) and DFS (HR = 0.48; 95% CI, 0.21–1.06).

### CD45R0+ Treg subset

**Figure 6:**
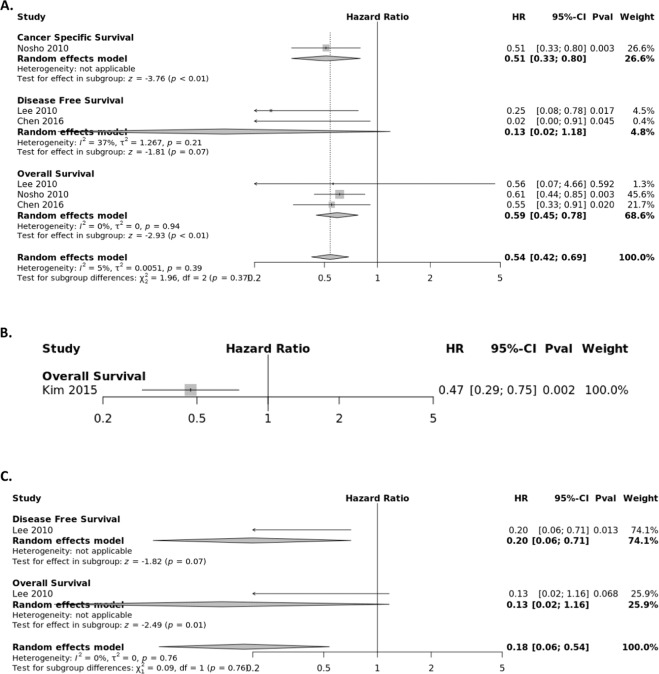
Forest plots of random effects between levels of CD45R0+ T-cell infiltrate and Survival. The effect of CD45R0+ T-cells in the (**A**) tumor center (**B**) invasive margin (**C**) stroma on cancer-specific survival (CSS), disease-free survival (DFS), and overall survival (OS).

The association between the presence of CD45R0+ T lymphocytes and survival of CRC patients was extracted from four studies (Fig. 6) stratified by tumor location, with three assessing the CC, one the TS, and one the IM. The pooled HR from the tumor center panel were calculated for OS (HR = 0.59; 95% CI, 0.45–0.78), CSS (HR = 0.51; 95% CI, 0.33–0.80) and DFS (HR = 0.13; 95% CI, 0.02–1.18). Estimated HRs for OS and DFS for CD45R0 + lymphocyte infiltrates from the invasive margin and tumor stroma were provided from single studies.

## Discussion

We have performed a systematic review and quantitative meta-analysis of the prognostic impact of tumor infiltrating lymphocyte density and composition on CRC outcome. Through a computerized, systematic literature search of Medline, Embase, Web of Science, and Scopus databases using pre-determined inclusion criteria, we identified 43 studies published between August 1997 and April 2017 (representing a total of 21,015 CRC patients with available samples) that evaluate specific marker subset populations of tumor infiltrating lymphocytes in CRC and survival outcomes. We separately considered Generalized TIL density, CD3, CD8, FOXP3, CD45R0 as the focus of our meta-analysis, recognizing that there are other systems of scoring the host immune response that are beyond the scope of the current meta-analysis. Since the publication of an initial meta-analysis of TILs and CRC in 2014 by Mei *et al*. which included 7840 patients, there have been an additional 13,175 CRC patients with tissue samples that have undergone analysis for TIL density by T-cell subset and histopathologic location. Due to the increasing recognition of intratumoral adaptive immune reaction as a prognostic marker for survival and as a therapeutic target of immune checkpoint inhibitors, we performed an updated systematic review and meta-analysis of TIL.

Pooled analysis from an extensive compilation of studies suggest that high generalized TIL counts and CD3+ T-cell density have the strongest association with survival benefit for patients as compared to low generalized TIL counts and CD3+ T-cell density in regards to disease-free survival (DFS), cancer-specific survival (CSS), and overall survival (OS). The pooled summary HRs for each T-cell subset were inconsistent across different studies. Some markers trended towards a stronger prognostic association with survival as compared to the earlier analysis performed by Mei *et al*. (CD3, CD8, FOXP3).

The effect of the immune system in colorectal cancer is still being elucidated as several prospective and retrospective studies demonstrate that robust antitumor immunity is associated with favorable prognosis in patients with CRC. Notably, we confirmed in our study a prognostic benefit of FoxP3+ T cell infiltrates which stands in contrast to previous meta-analyses suggesting that tumor-infiltrating FoxP3+ T-cells are associated with poor clinical outcomes in solid cancers^68,69^. Recent studies elucidating the interplay between the tumor microenvironment and colonic microbiome have identified two distinct subpopulations of immunosuppressive and proinflammatory FOXP3+ T-cells. Investigators found that proinflammatory FoxP3^lo^ T-cells were associated with an increased presence of *Fusobacterium nucleatum* and better CRC patient prognosis, while immunosuppressive FOXP3+ T-cellls were associated with worse outcomes^70^. Additional TIL research is ongoing in understanding the modulation of T-cell trafficking by the gut microbiome and the control of tumor growth through direct lysis of cancer cells through the production of cytokines that promote a cytotoxic response^71,72^. In addition, new immunotherapies are being developed that harness adoptive transfer of marker-specific TIL populations to elicit an immune response to tumors^73^.

Our meta-analysis demonstrates that generalized TIL density is a strong prognostic marker for survival in patients with colorectal cancer. This result is concordant with previous studies that identified the association of TILs with increased survival^74^. The strengths of the study include the addition of large retrospective studies by Rozek *et al*.^63^, and Sinicrope *et al*.^64^, which included 2,369 patients and 2,293 patients respectively, adding further precision and generalizability to the recognition that TILs confer a prognostic advantage with a maximum likelihood HR = 0.65 for overall survival.

These findings are consistent with previous meta-analyses^18^, yet our results have caveats that are relevant to this type of summary analysis. Heterogeneity existed in most analyses even though subgroup and overall summary estimates were similar. Also, studies that utilize different methods of TIL identification, small populations, and variations associated with archival specimens were pooled. Nonetheless, the more homogeneous TIL density summary estimates were similar to the overall summary estimates, suggesting that the overall summary measures are a reasonable estimation of prognosis associated with TILs. Second, the meta-analysis was subject to detection, verification and spectrum biases from the original studies. We may have overlooked relevant studies with results (negative or limited) that would modify the estimates. In addition, the different cutoff values for designation of high vs low TIL was a source of bias for this meta-analysis. Among the analyzed studies, the cutoff values included presence or absence (Nagtegaal *et al*.^28^; Cianchi *et al*.^30^; Gao *et al*.^32^; Ogino *et al*.^37^; Richards *et al*.^50,57^), TIL count with a different threshold for high vs low (Lee *et al*.^44^; Rozek *et al*.^63^), and mean, media, and quartiles (Naito et al.^25^; Guidoboni *et al*.^27^; Chiba *et al*.^31^; Menon *et al*.^33^; Galon *et al*.^14^; Salama *et al*.^39^; Frey *et al*.^43^; Lee *et al*.^44^; Nosho *et al*.^45^; Sinicrope *et al*.^40,64^; Yoon *et al*.^51^; Di Caro *et al*.^54^). Some studies detected TILs by tissue microarray while others used full histologic sections. These differences could be responsible for the variability in reaching a standardized method of TIL evaluation. Galon *et al*. along with other groups including Robins *et al*.^75^ are making efforts to develop standardized methods to evaluate TILS in order to improve consistency and reproducibility of TIL measurements for future diagnostic studies, yet these techniques have not been broadly adapted enough to summarize with meta-analysis of these specific approaches^76^. Given our results and the extensive literature demonstrating the intratumoral immune cell infiltrate as a highly informative prognostic indicator, further studies are warranted towards the goal of optimizing tumor classification and cancer staging.

## Supplementary information

Supplementary Tables.
