# Supplementary material for: The Prognostic Implications of Tumor Infiltrating Lymphocytes in Colorectal Cancer: A Systematic Review and Meta-Analysis
Source: Sci Rep. 2020 Feb 25;10:3360. doi: 10.1038/s41598-020-60255-4 (PMC7042281; doi:10.1038/s41598-020-60255-4)
Supplement: Supplementary file 1 — Supplementary Tables. [file 41598_2020_60255_MOESM1_ESM.docx]

**SUPPLEMENTARY INFORMATION**

**TITLE: The Prognostic Implications of Tumor Infiltrating Lymphocytes in Colorectal Cancer: A Systematic Review and Meta-Analysis.**

**Authors:** Gregory E. Idos MD, MS^1*^, Janet Kwok MD^2^, Nirupama Bonthala MD^2^, Lynn Kysh MLIS^2^, Stephen B. Gruber MD, PhD, MPH^1^, Chen Xu Qu PhD^2^

Supplementary Table 1. Study Quality Assessment

Supplementary Appendices: Search Terms Appendices

**Table S1. Study Quality Assessment**

| Study | Is the population under study defined with in- and exclusion criteria? | Were patient data prospectively collected? | Are the main prognostic patient and tumor characteristics presented?^1^ | Is the method used for determination of protein expression specified? | Is the IHC or HE staining protocol specified?^2^ | Were stainings evaluated by > 1 observer? | Is the study endpoint defined? | Is the time of follow up specified? | Is loss during analysis or follow up described? | Quality rating |
| --- | --- | --- | --- | --- | --- | --- | --- | --- | --- | --- |
| Ropponen et al 1997 | **0** | **0** | **1** | **0** | **1** | **1** | **1** | **1** | **0** | 5 |
| Naito et al  1998 | **0** | **0** | **0** | **1** | **1** | **Not specified** | **0** | **1** | **0** | 3 |
| Nielsen et al  1999 | **0** | **0** | **1** | **1** | **1** | **0** | **1** | **1** | **1** | 6 |
| Guidoboni et al 2001 | **0** | **0** | **1** | **0** | **1** | **1** | **1** | **1** | **0** | 5 |
| Nagtegaal et al 2001 | **0** | **0** | **0** | **1** | **1** | **Not specified** | **1** | **1** | **0** | 4 |
| Paraf et al  2001 | **0** | **0** | **1** | **1** | **1** | **0** | **1** | **1** | **0** | 5 |
| Cianchi et al  2002 | **1** | **0** | **1** | **0** | **1** | **Not clear** | **0** | **1** | **0** | 4 |
| Chiba et al  2004 | **1** | **0** | **1** | **1** | **1** | **1** | **0** | **1** | **0** | 6 |
| Menon et al  2004 | **0** | **0** | **1** | **1** | **1** | **Not Specified** | **0** | **1** | **0** | 4 |
| Table S1. Study Quality Assessment (Continued) | | | | | | | | | | |
| Study | **Is the population under study defined with in- and exclusion criteria?** | **Were patient data prospectively collected?** | **Are the main prognostic patient and tumor characteristics presented?^1^** | **Is the method used for determination of protein expression specified?** | **Is the IHC or HE staining protocol specified?^2^** | **Were stainings evaluated by > 1 observer?** | **Is the study endpoint defined?** | **Is the time of follow up specified?** | **Is loss during analysis or follow up described?** | Quality rating |
| Prall et al  2004 | **1** | **0** | **1** | **1** | **1** | **0** | **1** | **1** | **1** | 7 |
| Buckowitz et al 2005 | **1** | **1** | **0** | **1** | **1** | **1** | **0** | **1** | **0** | 6 |
| Gao et al  2005 | **0** | **0** | **1** | **1** | **1** | **1** | **0** | **1** | **0** | 5 |
| Klintrup et al 2005 | **0** | **0** | **1** | **1** | **0** | **1** | **1** | **1** | **1** | 6 |
| Galon et al  2006 | **1** | **0** | **1** | **1** | **1** | **Not specified** | **1** | **1** | **1** | 7 |
| Ogino et al  2009 | **1** | **1** | **1** | **1** | **0** | **1** | **1** | **1** | **0** | 7 |
| Roxburgh et al 2009 | **1** | **1** | **1** | **1** | **0** | **1** | **1** | **1** | **1** | 8 |
| Salama et al  2009] | **0** | **0** | **1** | **1** | **1** | **Not Specified** | **1** | **1** | **0** | 6 |
| Sinicrope et al 2009 | **0** | **0** | **0** | **1** | **1** | **0** | **1** | **1** | **0** | 4 |
| Table S1. Study Quality Assessment (Continued) | | | | | | | | | | |
| Study | **Is the population under study defined with in- and exclusion criteria?** | **Were patient data prospectively collected?** | **Are the main prognostic patient and tumor characteristics presented?^1^** | **Is the method used for determination of protein expression specified?** | **Is the IHC or HE staining protocol specified?^2^** | **Were stainings evaluated by > 1 observer?** | **Is the study endpoint defined?** | **Is the time of follow up specified?** | **Is loss during analysis or follow up described?** | Quality rating |
| Correale et al 2010 | **1** | **1** | **0** | **1** | **1** | **0** | **0** | **1** | **0** | 5 |
| Deschoolmeester et al  2010 | **0** | **0** | **1** | **1** | **1** | **1** | **0** | **1** | **1** | 6 |
| Frey et al  2010 | **0** | **0** | **1** | **1** | **1** | **Not specified** | **0** | **0** | **1** | 4 |
| Lee et al  2010 | **1** | **0** | **0** | **1** | **1** | **0** | **0** | **1** | **0** | 4 |
| Nosho et al  2010 | **0** | **1** | **1** | **1** | **1** | **0** | **1** | **1** | **0** | 6 |
| Peng et al  2010 | **0** | **0** | **1** | **1** | **1** | **1** | **1** | **1** | **1** | 6 |
| Simpson et al 2010 | **1** | **0** | **1** | **1** | **1** | **1** | **1** | **1** | **0** | 7 |
| Dahlin et al  2011 | 0 | 0 | 1 | 1 | 1 | 1 | 1 | 1 | 0 | 6 |
| \| Table S1. Study Quality Assessment (Continued) \| \| \| \| \| \| \| \| \| \| \| \| --- \| --- \| --- \| --- \| --- \| --- \| --- \| --- \| --- \| --- \| --- \| \| Study \| **Is the population under study defined with in- and exclusion criteria?** \| **Were patient data prospectively collected?** \| **Are the main prognostic patient and tumor characteristics presented?^1^** \| **Is the method used for determination of protein expression specified?** \| **Is the IHC or HE staining protocol specified?^2^** \| **Were stainings evaluated by > 1 observer?** \| **Is the study endpoint defined?** \| **Is the time of follow up specified?** \| **Is loss during analysis or follow up described?** \| Quality rating \| \| Huh et al  2012 \| **1** \| **0** \| **1** \| **1** \| **0** \| **1** \| **1** \| **1** \| 0 \| 6 \| \| Richards et al 2012 \| **1** \| **0** \| **1** \| **1** \| **1** \| **1** \| **1** \| **1** \| 0 \| 7 \| \| Yoon et al  2012 \| **0** \| **0** \| **1** \| **1** \| **1** \| **1** \| **1** \| **1** \| 0 \| 6 \| \| Kim et al  2013 \| **1** \| **0** \| **1** \| **1** \| **1** \| **1** \| **1** \| **1** \| 1 \| 8 \| \| Lewis et al  2013 \| **1** \| **0** \| **1** \| **0** \| **1** \| **1** \| **1** \| **1** \| 1 \| 7 \| \| Di Caro et al  2014 \| **1** \| **0** \| **1** \| **1** \| **1** \| **0** \| **1** \| **1** \| 1 \| 7 \| \| Ling et al  2014 \| **1** \| **0** \| **1** \| **1** \| **1** \| **0** \| **1** \| **1** \| 1 \| 7 \| \| Reimers et al 2014 \| **1** \| **0** \| **1** \| **1** \| **1** \| **Not specified** \| **1** \| **0** \| 0 \| 5 \| \| Table S1. Study Quality Assessment (Continued) \| \| \| \| \| \| \| \| \| \| \| \| Study \| **Is the population under study defined with in- and exclusion criteria?** \| **Were patient data prospectively collected?** \| **Are the main prognostic patient and tumor characteristics presented?^1^** \| **Is the method used for determination of protein expression specified?** \| **Is the IHC or HE staining protocol specified?^2^** \| **Were stainings evaluated by > 1 observer?** \| **Is the study endpoint defined?** \| **Is the time of follow up specified?** \| **Is loss during analysis or follow up described?** \| Quality rating \| \| Richards et al 2014 \| **1** \| **1** \| **1** \| **1** \| **1** \| **1** \| **1** \| **1** \| 0 \| 8 \| \| Kim et al  2015] \| **0** \| **1** \| **1** \| **1** \| **1** \| **1** \| **1** \| **1** \| 1 \| 8 \| \| Mori et al  2015 \| **1** \| **0** \| **1** \| **1** \| **1** \| **Not specified** \| **1** \| **1** \| 0 \| 6 \| \| Reissfelder et al 2015 \| **1** \| **0** \| **1** \| **1** \| **0** \| **1** \| **1** \| **1** \| 1 \| 7 \| \| Vlad et al  2015 \| **1** \| **0** \| **1** \| **0** \| **1** \| **0** \| **1** \| **1** \| 0 \| 5 \| \| Wang et al  2015 \| **1** \| **0** \| **1** \| **1** \| **1** \| **0** \| **1** \| **1** \| 0 \| 6 \| \| Rozek et al  2016 \| **0** \| **1** \| **1** \| **0** \| **1** \| **0** \| **1** \| **1** \| 1 \| 6 \| \| Sinicrope et al 2016 \| **0** \| **0** \| **1** \| **1** \| **1** \| **0** \| **1** \| **1** \| 0 \| 5 \| \| Chen et al  2016 \| 0 \| 0 \| 1 \| 1 \| 1 \| 0 \| 1 \| 1 \| 0 \| 0 \| | | | | | | | | | | |

Abbreviations: IHC = immunohistochemistry; HE staining=Hematoxylin-eosin staining

^1^At least four of the following characteristics: age at diagnosis, stage, tumor type, differentiation grade. ^2^At least four of the following criteria: antigen retrieval, primary antibody, dilution, detection method, cut-off value for positive expression.

**Supplementary Appendix:**

**Appendix 1: MEDLINE (PubMed)**

1946 – April 6, 2017

1. Colorectal Neoplasms[Mesh]

2. Colon[Mesh]

3. Rectum[Mesh]

4. colorectal

5. colo-rectal

6. colonic

7. colon

8. rectum

9. rectal

10. #2-#9/OR

11. Neoplasms[Mesh]

12. neoplasm

13. neoplasms

14. neoplasia

15. tumor

16. tumors

17. tumour

18. tumours

19. carcinoma

20. carcinomas

21. cancer

22. cancers

23. adenoma

24. adenomas

25. adenocarcinoma

26. adenocarcinomas

27. adenomatous

28. #11-#27/OR

29. #10 AND #28

30. #1 OR #29

31. Lymphocytes, Tumor-Infiltrating[Mesh]

32. T-Lymphocytes[Mesh]

33. T-Lymphocytes, Cytotoxic[Mesh]

34. Receptors, CCR7[Mesh]

35. t-lymphocyte

36. t-lymphocytes

37. TIL

38. TILS

39. ccr7 receptor

40. ccr7 receptors

41. #31-#40/OR

42. tumor infiltrating

43. tumor-infiltrating

44. tumor derived

45. tumor-derived

46. tumour infiltrating

47. tumour-infiltrating

48. tumour derived

49. tumour-derived

50. #42-#49/OR

51. Lymphocytes[Mesh]

52. lymphocyte

53. lymphocytes

54. lymphoid cell

55. lymphoid cells

56. activated cell

57. activated cells

58. #51-#57/OR

59. #50 AND #58

60. #41 OR #59

61. Prognosis[Mesh]

62. Mortality[Mesh]

63. Survival Analysis[Mesh]

64. Cohort Studies[Mesh]

65. prognosis

66. prognoses

67. disease free survival

68. disease free survival

69. medical futility

70. neoplasm grading

71. neoplasms grading

72. nomograms

73. treatment outcome

74. mortality

75. mortalities

76. cause of death

77. fatal outcome

78. survival rate

79. survival analyses

80. Kaplan-meier estimate

81. cohort study

82. cohort studies

83. cohort analysis

84. cohort analyses

85. concurrent study

86. concurrent studies

87. longitudinal study

88. longitudinal studies

89. follow up study

90. follow up studies

91. prospective study

92. prospective studies

93. #61-#92/OR

94. #30 AND #60 AND #93

English Language Filter Applied

**Appendix 2: Embase (Ovid)**

1970 – October 2, 2015

1. ‘Colon Cancer’/exp

2. ‘Rectum Tumor’/exp

3. #1 OR #2

4. ‘Colon’/exp

5. ‘Rectum’/exp

6. colorectal.kw

7. colo-rectal.kw

8. colonic.kw

9. colon.kw

10. rectum.kw

11. rectal.kw

12. #4-#11/OR

13. ‘Neoplasm’/exp

14. neoplasms.kw

15. neoplasms.kw

16. neoplasia.kw

17. tumor.kw

18. tumors.kw

19. tumour.kw

20. tumours.kw

21. carcinoma.kw

22. carcinomas.kw

23. cancer.kw

24. cancers.kw

25. adenoma.kw

26. adenomas.kw

27. adenocarcinoma.kw

28. adenocarcinomas.kw

29. adenomatous.kw

30. #13-#30/OR

31. #12 AND #30

32. #30 OR #31

33. ‘Tumor Associated Leukocyte’/exp

34. ‘T Lymphocyte’/exp

35. ‘Cytotoxic Lymphocyte’/exp

36. ‘Chemokine Receptor CCR7’/exp

37. (t lymphocyte) .kw

38. (t lymphocytes) .kw

39. TIL.kw

40. TILS.kw

41. (CCR7 receptor) .kw

42 (CCR7 receptors) .kw

43. #33-#42/OR

44. (tumor infiltrating) .kw

45. (tumor derived) .kw

46. (tumour infiltrating) .kw

47. (tumour derived) .kw

48. #44-#47/OR

49. ‘Lymphocyte’/exp

50. lymphocyte.kw

51. lymphocytes .kw

51. (lymphoid cells).kw

52. (lymphoid cell).kw

53. (activated cell).kw

54. (activated cells).kw

55. #49-#54/OR

56. (activated cell).kw

57. (activated cells).kw

58. #51-#57/OR

59. #48 AND #58

60. #43 OR #59

61. ‘Prognosis’/exp

62. ‘Mortality’/exp

63. ‘Survival’/exp

64. ‘Cohort Analysis’/exp

65. ‘Disease Free Survival’/exp

66. ‘Treatment Outcome’/exp

67. ‘Cancer Grading’/exp

68. ‘Nomogram’/exp

69. ‘Cause of Death’/exp

70. ‘Fatality’/exp

71. ‘Survival Rate’exp

72. ‘Kaplan Meier Method’/exp

73. ‘Longitudinal Study’/exp

74. ‘Prospective Study’/exp

75. prognosis.kw

76. prognoses.kw

77. (disease free survival).kw

78. (neoplasm grading).kw

79. (neoplasm staging).kw

80. nomograms.kw

81. (treatment outcome).kw

82. mortality.kw

83. mortalities.kw

84. (cause of death).kw

85. (fatal outcome).kw

86. fatality.kw

87. fatalities.kw

88. (survival rate).kw

89. (survival analysis).kw

90. (survival analyses).kw

91. (Kaplan-Meier).kw

92. (cohort study).kw

93. (cohort studies).kw

94. (cohort analysis).kw

95. (cohort analyses).kw

96. (concurrent study).kw

97. (concurrent studies).kw

98. (longitudinal study).kw

99. (longitudinal studies).kw

100. (follow up study).kw

101. (follow up studies).kw

102. (prospective study).kw

103. (prospective studies).kw

104. #61-#103/OR

105. #32 AND #60 AND #104

English Language Filter Applied

Removed MEDLINE Records

**Appendix 3: Embase (Elsevier)**

1946 – April 6, 2017

1. ‘Colon Cancer’/exp

2. ‘Rectum Tumor’/exp

3. #1 OR #2

4. ‘Colon’/exp

5. ‘Rectum’/exp

6. colorectal

7. colo-rectal

8. colonic

9. colon

10. rectum

11. rectal

12. #4-#11/OR

13. ‘Neoplasm’/exp

14. neoplasms

15. neoplasms

16. neoplasia

17. tumor

18. tumors

19. tumour

20. tumours

21. carcinoma

22. carcinomas

23. cancer

24. cancers

25. adenoma

26. adenomas

27. adenocarcinoma

28. adenocarcinomas

29. adenomatous

30. #13-#30/OR

31. #12 AND #30

32. #30 OR #31

33. ‘Tumor Associated Leukocyte’/exp

34. ‘T Lymphocyte’/exp

35. ‘Cytotoxic Lymphocyte’/exp

36. ‘Chemokine Receptor CCR7’/exp

37. (t lymphocyte)

38. (t lymphocytes)

39. TIL

40. TILS

41. (CCR7 receptor)

42 (CCR7 receptors)

43. #33-#42/OR

44. (tumor infiltrating)

45. (tumor derived)

46. (tumour infiltrating)

47. (tumour derived)

48. #44-#47/OR

49. ‘Lymphocyte’/exp

50. lymphocyte

51. lymphocytes

51. (lymphoid cells)

52. (lymphoid cell)

53. (activated cell)

54. (activated cells)

55. #49-#54/OR

56. activated cell

57. activated cells

58. #51-#57/OR

59. #48 AND #58

60. #43 OR #59

61. ‘Prognosis’/exp

62. ‘Mortality’/exp

63. ‘Survival’/exp

64. ‘Cohort Analysis’/exp

65. ‘Disease Free Survival’/exp

66. ‘Treatment Outcome’/exp

67. ‘Cancer Grading’/exp

68. ‘Nomogram’/exp

69. ‘Cause of Death’/exp

70. ‘Fatality’/exp

71. ‘Survival Rate’exp

72. ‘Kaplan Meier Method’/exp

73. ‘Longitudinal Study’/exp

74. ‘Prospective Study’/exp

75. prognosis

76. prognoses

77. (disease free survival)

78. (neoplasm grading)

79. (neoplasm staging)

80. nomograms

81. (treatment outcome)

82. mortality

83. mortalities

84. (cause of death)

85. (fatal outcome)

86. fatality

87. fatalities

88. (survival rate)

89. (survival analysis)

90. (survival analyses)

91. (Kaplan-Meier)

92. (cohort study)

93. (cohort studies)

94. (cohort analysis)

95. (cohort analyses)

96. (concurrent study)

97. (concurrent studies)

98. (longitudinal study)

99. (longitudinal studies)

100. (follow up study)

101. (follow up studies)

102. (prospective study)

103. (prospective studies)

104. #61-#103/OR

105. #32 AND #60 AND #104

English Language Filter Applied

**Appendix 4: Cochrane Library (Wiley)**

Issue 4 of 12, April 2017 – Searched April 6, 2017

(Title, Abstract, Keyword)

1. colorectal

2. colonic

3. colon

4. rectum

5. rectal

6. #1-#5/OR

7. neoplasms

8. neoplasm

9. neoplasia

10. tumor

11. tumors

12. tumour

13. tumours

14. carcinoma

15. carcinomas

16. cancer

17. cancers

18. adenoma

19. adenomas

20. adenocarcinoma

21. adenocarcinomas

22. adenomatous

23. #7-#22/OR

24. #6 AND #23

25. t-lymphocyte

26. t-lymphocytes

27. ccr7 receptor

28. ccr7 receptors

29. TIL

30. TILS

31. #25-#30/OR

32. tumor infiltrating

33. tumor-infiltrating

34. tumor derived

35. tumor-derived

36. tumour infiltrating

37. tumour-infiltrating

38. tumour derived

39. tumour-derived

40. #32-#39/OR

41. lymphocytes

42. lymphocyte

43. lymphoid cell

44. lymphoid cells

45. activated cell

46. activated cells

47. #41-#46/OR

48. #40 AND #47

49. #31 OR #48

50. prognosis

51. prognoses

52. mortality

53. survival analysis

54. disease free survival

55. medical futility

56. neoplasm grading

57. neoplasm staging

58. nomograms

59. treatment outcome

60. mortalities

61. cause of death

62. fatal outcome

63. survival rate

64. survival analysis

65. survival analyses

66. Kaplan-meier

67. #50-#66/OR

68. #24 AND #49 AND #67

**Appendix 5: Web of Science (Thomson-Reuters)**

1900 – April 6, 2017

1. TS=(colorectal)

2. TS=(colo-rectal)

3. TS=(colonic)

4. TS=(colon)

5. TS=(rectum)

6. TS=(rectal)

7. #1-#6/OR

8. TS=(neoplasm)

9. TS=(neoplasia)

10. TS=(tumor)

11. TS=(tumour)

12. TS=(carcinoma)

13. TS=(cancer)

14. TS=(adenoma)

15. TS=(adenocarcinoma)

16. TS=(adenomatous)

17. #8-#16/OR

18. #7 AND #17

19. TS=(t-lymphocyte)

20. TS=(t-lymphocytes)

21. TS=(TIL)

22. TS=(TILS)

23. TS=(CCR7 receptor)

24. #18-#22/OR

25. TS=(tumor infiltrating)

26. TS=(tumor-infilitrating)

27. TS=(tumor derived)

28. TS=(tumor-derived)

29. TS=(tumour infiltrating)

30. TS=(tumour-infiltrating)

31. TS=(tumour derived)

32. TS=(tumour-derived)

33. #24-#31/OR

34. TS=(lymphocyte)

35. TS=(lymphoid cell)

36. TS=(activated cell)

37. #33-#35/OR

38. #33 AND #37

39. #24 OR #38

40. TS=(prognosis)

41. TS=(disease free survival)

42. TS=(disease-free survival)

43. TS=(medical futility)

44. TS=(neoplasm grading)

45. TS=(neoplasm staging)

46. TS=(nomograms)

47. TS=(treatment outcome)

48. TS=(mortality)

49. TS=(cause of death)

50. TS=(fatal outcome)

51. TS=(survival rate)

52. TS=(survival analysis)

53. TS=(Kaplan-meier estimate)

54. TS=(cohort study)

55. TS=(cohort analysis)

56. TS=(concurrent study)

57. TS=(longitudinal study)

58. TS=(follow-up study)

59. TS=(prospective study)

60. #40-#59/OR

61. #18 AND #39 AND #60

English Language Filter Applied

**Appendix 6 - ClinicalTrials.gov (US National Institutes of Health)**

2000 – April 6, 2017

1. colorectal

2. colon

3. rectal

4. #1-#3/OR

5. cancer

6. neoplasm

7. #5-#6/OR

8. TILS

9. t-lymphocyte

10. tumor infiltrating lymphocyte

11. tumor derived lymphocyte

12. prognosis

13. survival

14. mortality

15. #8-#14/OR

16. #4 AND #7 AND #15
